# Comparison of Postoperative Complications after Endoscopic Submucosal Dissection: Differences of Insufflations and Anesthesias

**DOI:** 10.1155/2011/709237

**Published:** 2011-07-07

**Authors:** Hirohito Mori, Hideki Kobara, Akemi Muramatsu, Hideyuki Inoue, Mitsuyoshi Kobayashi, Takako Nomura, Masanobu Hagiike, Kunihiko Izuishi, Yasuyuki Suzuki, Jian Gong, Tsutomu Masaki

**Affiliations:** ^1^Department of Gastroenterology and Neurology, Kagawa Medical University School of Medicine, 1750-1 Ikenobe, Miki-cho, Kita-gun, Kagawa 761-0793, Japan; ^2^Department of Gastroenterological Surgery, Kagawa Medical University School of Medicine, 1750-1 Ikenobe, Miki-cho, Kita-gun, Kagawa 761-0793, Japan

## Abstract

Endoscopic submucosal dissection (ESD) has enabled the collective resection and increased the accuracy of pathological diagnosis. However, ESD requires a long operation time, which results in increased doses of analgesics/sedatives, and causes worsening of respiratory and hemodynamic statuses. To reduce postoperative complications, we have applied ESD with CO_2_ insufflation and general anesthesia. This study included 50 patients who underwent ESD for early gastric cancer, 25 with air insufflation and intravenous anesthesia (Air/IV group), and the remaining 25 with CO_2_ insufflation and general anesthesia (CO_2_/GA group). Postoperative enlarged feeling of the abdomen was observed only in 1 of 25 patients in the CO_2_/GA group (*P* = 0.0416). Postoperative severe unrest was observed in none of the patients in the CO_2_/GA group and in 4 of 25 (16%) patients in the Air/IV group (*P* = 0.0371). CO_2_ insufflation and general anesthesia are useful in stabilizing intraoperative conditions and reducing postoperative complications.

## 1. Introduction

Endoscopic submucosal dissection (ESD) for gastrointestinal malignancy has spread across Japan, with its technical basis almost completely established. The procedure is also covered by the health insurance system in Japan [1, 2]. However, there is a large gap in ESD skill levels between institutions. Intraoperative analgesic/sedative regimens also vary among institutions. There are several recent studies on ESD under intravenous (IV) propofol anesthesia using a bispectral index (BIS) monitor [3, 4]. However, therapeutic endoscopy lasting for 120 minutes or longer is no longer low-invasive from the perspective of patient safety management [5, 6] and thus requires systemic management, including respiratory/cardiovascular management, as in the case of laparoscopic surgery. In addition, with an increasingly aging patient population, the possibilities of complications such as brain/cardiac disorders and pulmonary embolism, as well as ESD-associated accidental events, such as bleeding and perforation, should be considered for safety reasons. In case of emergency, such as when any of the above complications has occurred, patient management performed with IV anesthesia alone in an endoscopy room is inadequate, and ESD performed by an anesthesiologist under general anesthesia is essentially required for the early detection and immediate treatment of complications. In addition, for a favorable postoperative quality of life (QOL) of patients, CO_2_ insufflation is very useful for avoiding an abnormal accumulation of air in the gastrointestinal (GI) tract [7–12]. While there have been several reports of ESD performed with CO_2_ insufflation and general anesthesia, no study has compared postoperative complications following ESD with CO_2_ insufflation and general anesthesia with those following ESD with air insufflation and IV anesthesia. This study demonstrates the usefulness of ESD with CO_2_ insufflation and general anesthesia in reducing postoperative complications compared to ESD with air insufflation and IV anesthesia.

## 2. Materials and Methods

This study included 50 patients (43 males and 7 females) who underwent gastric ESD for the treatment of early gastric cancer at Kagawa Rosai Hospital or Kagawa University Hospital between June 2007 and March 2010. 

**Figure 1:**
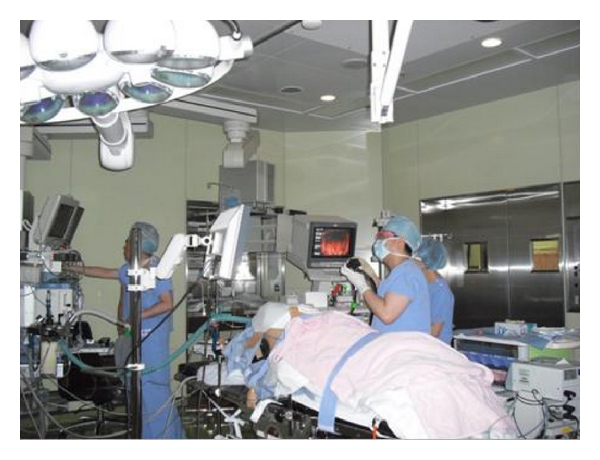
A view of ESD with CO_2_ insufflation and general anesthesia at Kagawa University Hospital. General anesthesiais performed by an anesthesiologist.

Of the 50 patients, 25 underwent ESD with air insufflation and IV anesthesia (Air/IV group) and the remaining 25 underwent ESD with CO_2_ insufflation and general anesthesia (CO_2_/GA group) (Figure 1). We selected CO_2_/GA group by following cases. The procedure time is over 120 min, the size of the tumor is over 30 mm in diameter, and the location of the lesion is difficult to be treated such as fornix, upper body of the stomach.

All patients were performed by one endoscopist who performed 150 gastric ESD cases per year. In this study, all ESDs were performed by single operator.

### 2.1. Background of Patients

Patient background is summarized in Table 1. There were no significant differences between groups in age, sex, operation time, location of lesion and diameter of lesion. 

Postoperative unrest was classified according to severity into mild and severe unrest. Patients with mild unrest were defined as being able to follow instructions and maintain a resting state and could be left unattended while those with severe unrest were defined as being unable to follow instructions or able to follow instructions but immediately repeat the same behaviors and unable to maintain a resting state, requiring the attendance of an observer in a treatment room throughout the night. The severity of unrest was determined by a nurse after the patient returned to a patient room. In case of severe unrest, the attending physician was notified and a patient's family member was required to stay with the patient. We used the ASA conscious levels for references. The guideline of the American Society of Anesthegiology (ASA) divides into four levels like the following:

  (1) minimal sedation,
  (2) moderate sedation and analgesia (conscious sedation),
  (3) deep sedation and analgesia,
  (4) general anesthesia.

We defined mild unrest as the conscious level of (1), and severe unrest as (2) to (3).

The incidences of postoperative nausea, vomiting, enlarged feeling of the abdomen, and unrest (mild and severe) were retrospectively compared between the two groups. 

In our hospital, the postoperative bleeding rate was about 0.15% on average from April 2007 to March 2010.

### 2.2. Statistical Analysis

The chi-square test was used for between-group comparisons, with a significance level of *P* < 0.05.

## 3. Results

### 3.1. Comparison of the Incidences of Postoperative Complications between Groups

Postoperative nausea/vomiting was observed in 5 of 25 patients in the CO_2_/GA group, with an incidence of about 20%, and in 6 of 25 (about 24%) patients in the Air/IV group. A 2 × 2 chi-square test showed no significant difference between the two groups (*P* = 0.7328) (Table 2). 

Postoperative enlarged feeling of the abdomen was observed in 1 of 25 patients in the CO_2_/GA group, with an incidence of about 4%, and in 6 of 25 (about 24%) patients in the Air/IV group. A 2 × 2 chi-square test revealed a significant difference between the two groups (*P* = 0.0416) (Table 2). 

Postoperative mild unrest was observed in 4 of 25 (16%) patients in the CO_2_/GA group and in 3 of 25 (12%) patients in the Air/IV group, with no significant difference between groups by a 2 × 2 chi-square test (*P* = 0.6836) (Table 2). 

Postoperative severe unrest was observed in none of the patients in the CO_2_/GA group and in 4 of 25 (16%) patients in the Air/IV group, with a significant difference between groups by a 2 × 2 chi-square test (*P* = 0.0371) (Table 2).

## 4. Discussion

In Japan, an increasing life expectancy has also led to the aging of the population of patients undergoing cancer treatment. This also applies to patients undergoing ESD for treatment of early gastric cancer. ESD is therefore actively performed in elderly patients. However, elderly patients are more likely to develop serious complications during and after the procedure than younger patients [1, 2, 4]. These complications include intraoperative worsening of respiratory/hemodynamics status, perforation caused by abrupt body movement, postoperative abdominal symptoms, and unrest. It is therefore important to develop safer methods of intraoperative management and gas insufflation during ESD for the treatment of early gastric cancer [3, 5, 6]. In the present study, we compared the incidence of postoperative abdominal symptoms and severity of unrest following ESD with CO_2_ insufflation and general anesthesia with those following ESD with air insufflation and IV anesthesia for the treatment of early gastric cancer. While there were no significant differences in age, sex, location of lesion, diameter of lesion, or operation time between the two groups, the severity of postoperative abdominal symptoms was significantly milder and the incidence of severe unrest was significantly lower following ESD with CO_2_ insufflation and general anesthesia. These results indicate that ESD with CO_2_ insufflation and general anesthesia is safer than ESD with air insufflation and IV anesthesia for the treatment of early gastric cancer. Other advantages of using ESD with general anesthesia for the treatment of early gastric cancer include the absence of risks of respiratory depression or aspiration due to intratracheal intubation [3–5]. It also allows a surgeon to delegate the intraoperative circulatory/respiratory management to an anesthesiologist so that he/she can concentrate on surgery [3]. 

Originally, elderly patients have often more serious complications of respiratory diseases or cardiovascular diseases. The respiratory or cardiovascular complication risks of elderly patients during the procedure are very high, and it is necessary to be monitored in elderly patients' condition by anesthegiologist. As early reduction of abdominal gas might reduce the abdominal complications such as abdominal compartment syndrome, we would better to use the CO_2_ insufflation.

In conclusion, ESD with CO_2_ insufflation and general anesthesia is safer than ESD with air insufflation and IV anesthesia. CO_2_ insufflation and general anesthesia should be used especially when performing ESD in elderly patients.

##  Conflict of Interests

The authors declare no conflicts of interest and no financial arrangement with any company.

## Figures and Tables

**Table 1:** 

	Air insufflation/IV anesthesia *n* = 25	CO_2_ insufflation/general anesthesia *n* = 25	*P* value
Age	73.2 ± 7.6 (65~88)	74.2 ± 6.7 (65~88)	NS*
Sex (male/female)	23/2	20/5	NS**
Operation time (min)	140.6 ± 74.1 (60~330)	163.4± 79.6 (80~420)	NS*
Location of lesion (U/M/L)	5/14/6	6/13/6	NS***
Diameter of lesion (mm)	39.9 ± 18.5 (13~90)	59.0 ± 17.6 (30~100)	NS*

*Mann-Whitney *U* test, **Fisher's exact test, ***Unpaired *t*-test.

**Table 2:** 

	Air insufflation/IV anesthesia *n* = 25	CO_2_ insufflation/general anesthesia *n* = 25	*X* ^2^ test (*P* value)
Postoperative nausea/vomiting	6	5	0.7328
Enlarged feeling of abdomen	6	1	0.0416 (<0.05)
Mild unrest	3	4	0.6836
Severe unrest	4	0	0.0371 (<0.05)
